# ADHD, ODD, and CD: Do They Belong to a Common Psychopathological Spectrum? A Case Series

**DOI:** 10.1155/2012/520689

**Published:** 2012-10-11

**Authors:** Sayanti Ghosh, Mausumi Sinha

**Affiliations:** Department of Psychiatry, R.G.Kar Medical College, West Bengal, Kolkata 700004, India

## Abstract

*Purpose of Research*. Numerous studies have reported comorbidities, overlapping symptoms, and shared risk factors among cases of attention deficit hyperactivity disorder (ADHD), oppositional defiant disorder (ODD) and conduct disorder (CD). We present three adolescent males aged 13–16 years with conduct disorder having past history of ADHD and ODD. *Principal Result*. The symptom profile especially in domains of aggression, hostility, and emotionality as well as the manner of progression from ADHD to ODD and CD in the above cases shows a similar pattern. *Conclusion*. These common developmental pathways and overlapping symptoms suggest the possibility of a common psychopathological spectrum encompassing the three externalizing disorders.

## 1. Introduction

Attention deficit hyperactivity disorder (ADHD), oppositional defiant disorder (ODD), and conduct disorder (CD) are three of the most prevalent disruptive behavior disorders in childhood and adolescence affecting approximately 1%–15% of all school age children and constituting a major proportion of referrals to mental health clinics [1, 2]. Numerous studies have reported comorbidities among these disorders both in epidemiological and clinical samples [2–5] often making them difficult to isolate and understand individually. In the following case reports we noticed a similar developmental pattern of these disorders. The diagnoses were made using the Structured Clinical Interview Schedule for the *Diagnostic and Statistical Manual* of Mental Disorders by experienced psychiatrists after obtaining consent from the subjects, their relatives maintaining anonymity and ethical considerations. Three adolescent patients presented with symptoms suggestive of conduct disorder with preceding history conducive to attention deficit hyperactivity disorder and oppositional defiant disorder according to Diagnostic and Statistical Manual of Mental Disorders (DSM-IV TR).

## 2. Case 1

A 14-year-old male student of ninth standard was brought with complaints of aggressiveness, disobedience, stealing, lying, truancy, frequent school fights, and deteriorating school grades for the past 3 years. Recently for last 6 months he had become irritable, throwing tantrums on minor issues, spending long hours in cyber cafes and self-refreshing along with like-minded peers in restaurants. He refused to attend school and often failed to complete the task assigned by the tutors for which there were repeated complaints from the school authorities. He was stealing money from his parents but they never interfered because of his assaultive nature. There was no history of brain trauma or seizures. 

Past history revealed hyperactivity at home from the age of 3 years and subsequently disturbances of activity and attention since 4 years of age during initial school years. This led to parental abuse in the growing years. Previous prescriptions revealed diagnosis of ADHD-combined type that improved partially with behavior therapy and parental counseling. Treatment was discontinued 2 years thereafter. Symptoms reappeared 3-4 years later with added defiant behavior, frequent temper tantrums, refusal to comply with rules in school and home. 

Family history revealed father using alcohol and marital disharmony between the parents. 

Birth and development milestones were normal; there was no history of febrile convulsions in his childhood. 

Mental state examination revealed guarded attitude towards the examiner. There was paucity of speech and affect was irritable. No thought or perceptual disturbances could be elicited. Higher cognitive functions were normal. 

Electroencephalography, MRI of brain, and blood biochemistry (including thyroid profile) were normal. 

IQ as tested by Wechsler intelligence scale for children (WISC-) IV full scale was found to be 95 (normal). 

Children's apperception test (CAT) revealed parental deprivation and hostile environment.

 He is being managed with Risperidone 3 mg at bedtime and Sodium valproate 800 mg per day in divided dosage along with behaviour therapy. On followup at 4 weeks and 3 months the boy has shown moderate improvement and is continuing with the management. 

## 3. Case 2

A 13-year-old male student studying in eighth grade presented with problems of inattentiveness, frequent school absenteeism, truancy, repeated failure in class with expulsion from a school in past, bullying classmates with frequent fights and destruction of school property, stealing money, compulsive internet surfing, and becoming violent if resisted. Exacerbation of these symptoms happened over last 6 months though he has been showing oppositional behavior for last 2 to 3 years in the form of disobedience, temper tantrums, rudeness, and refusal to study. He was also stealing money for buying fancy items and physically abused mother if resisted.

Past history revealed child being hyperactive since 6 years of age mostly pronounced at home. Subsequently there were repeated complaints from school regarding child's impulsivity and inattention. Gradually deteriorating scholastic performance and increasing defiant and uncontrollable behavior made psychiatric consultation mandatory when the child was around 9 years of age. He was diagnosed with ADHD-combined type and was treated with Methyl-phenidate 10 mg and behavior therapy, which was abruptly discontinued 6 months later. 

Family history reveals mother suffering from recurrent depressive disorder with hypothyroidism and father having bipolar-II disorder. Both of them are on medication. Paternal grandmother and uncle are also suffering from untreated bipolar disorder. 

Birth and developmental history are normal with no history of febrile convulsions.

Mental state examination revealed increased psychomotor activity, impulsivity, and obvious restlessness. Subjectively, the patient reported depressed mood, though his affect was irritable. Attention and concentration was impaired.

Investigations were normal. IQ (WISC-IV) was 90 (normal).

CAT reveals hostility and aggressiveness towards authority figures.

He is currently being treated with Atomoxetine 25 mg per day. Risperidone 2 mg per day has been added recently since his violent behavior and conduct problems have become pronounced. Behaviour therapy along with parental counseling is also being done. On followup in subsequent visits the boy has shown considerable improvement.

## 4. Case 3

A 16-year-old male student of class IX was brought with complaints of stealing money, excessive lying, setting fire to household items, teasing young girls of the locality, and passing lewd remarks and making obscene gestures. The onset was insidious 3 years back and has exacerbated over the past 8 months. Of late he has started using tobacco, cannabis, and alcohol and spending more time outdoors with friends of similar interest. Impulsivity and inattentiveness led to decline in scholastic performance and repetition of grades twice. Further school reports suggested gradual development of defiant behaviors such as openly defying rules in school, playing truant, instigating fellow students to pass silly remarks in class, disrespectful attitude towards elders ultimately resulting in frequent school absenteeism, mixing with local goons, and present conduct problems.

Past history reveals hyperactive behavior from the age of 3 years. Child was extremely prone to accidents right from childhood and had to be hospitalized on several occasions. In school he was impulsive and fidgety disturbing peers and failed to concentrate in his studies. 

No therapy was administered to the patient. 

Family history is disturbed; parents separated and mother had remarried. Relationship with stepfather was extremely hostile and there were frequent conflicts amongst the family members.

Birth and developmental milestones are normal.

Mental state examination shows signs of distractibility, an irritable affect, and impaired attention and concentration.

Blood biochemistry, EEG, and MRI brain were normal. IQ by WISC-IV was 95 (normal).

CAT reveals neglect and ignorance from parental figures associated with hostility towards them.

At present child is receiving Tab. Risperidone—4 mg daily and behavioral therapy is being done. On followup patient was found to be doing well.

## 5. Discussion

The developmental pathways from ODD to CD have been well studied with some researchers maintaining that ODD is relatively benign discrete disorder with good prognosis [6] and that ODD and CD follow a divergent course [7]. Other studies believe the two disorders to be hierarchically related with ODD to be a mild form of CD [8], with only a proportion of ODD cases progressing to CD [6, 9]. ODD emerged as a significant precursor of adolescent CD in children with ADHD independent of ADHD severity, the risk of CD being three times higher in such children [10]. Researchers who studied the role of ADHD symptoms and later development of CD have found that although ADHD can cooccur with CD, the association between ADHD and CD is largely accounted for by accompanying ODD [11]. In another study two subtypes of ODD associated with ADHD were identified: one that is prodromal to CD and another that is subsyndromal to CD but not likely to progress into CD in later years. These ODD subtypes have different correlates, course, and outcome [12]. All our cases indicate a common pattern of ADHD symptoms progressing to ODD and later development of CD with overlapping of symptoms at all stages.

It is known from twin studies that genetic influences are important in the development of ADHD symptoms [13–15], whereas both genes and shared environmental influences (i.e., nongenetic influences that contribute to cotwin similarity) seem to be important in the development of ODD [16] and CD symptoms [17, 18]. In addition, family studies have shown that ADHD, ODD, and CD are co-transmitted in families and thus appear to share a common familial etiology [5]. Genetic studies also indicate that CD was most strongly linked with attention deficit hyperactivity disorder-hyperactive-impulsive (ADHD-HI) type with a shared genetic heritability of 37%, ODD also strongly linked to attention deficit hyperactivity disorder-hyperactive-impulsive (ADHD-HI) with heritability of 42%. Both had lower correlations with both ADHD-combined type and ADHD-inattentive type [19]. All the cases reported past history of combined and hyperactive subtypes of childhood onset ADHD respectively. Regarding symptomatology, behavior problems associated with mood symptoms are a feature common to these disorders. Previous studies examining self-reported expression of overt aggressive behaviors and covert emotional and cognitive processes in adolescents diagnosed with ADHD and comorbid disruptive behavior disorders (DBDs) during childhood have reported emotional dysregulation to be an important component of ADHD along with inattention and hyperactivity [20]. This is similarly evident in our second case report.

So it appears that although there are meaningful distinctions among ADHD, ODD, and CD, especially in their correlates and outcomes [21], observation of their common vulnerabilities suggests that they share the same liability risk which could be either genetic, environmental, or some combination of both in origin. Keeping in mind these shared risk factors as well as overlapping symptoms especially in domains of aggression, hostility, and emotionality the developmental pathway from ADHD to ODD and CD in the above cases might raise the possibility of a common psychopathological spectrum. This needs to be further elucidated with larger sample sizes and detailed studies.
